# Enhancement of polysaccharides production using microparticle enhanced technology by *Paraisaria dubia*

**DOI:** 10.1186/s12934-021-01733-w

**Published:** 2022-01-28

**Authors:** Ling-Ling Tong, Yue Wang, Li Yuan, Meng-Zhen Liu, Yuan-Hang Du, Xin-Ya Mu, Qing-Hao Yang, Shi-Xiang Wei, Jun-Ya Li, Mian Wang, Dong-Sheng Guo

**Affiliations:** School of Food Science and Pharmaceutical Engineering, Nanjing Normal University, No. 1 Wenyuan Road, Nanjing, 210023 People’s Republic of China

**Keywords:** Cordyceps polysaccharides, *Paraisaria dubia*, Submerged fermentation, Morphological regulation

## Abstract

**Background:**

Polysaccharides are important active ingredients in *Ophiocordyceps gracilis* with many physiological functions. It can be obtained from the submerged fermentation by the anamorph (*Paraisaria dubia*) of *Ophiocordyceps gracilis*. However, it was found that the mycelial pellets of *Paraisaria dubia* were dense and increased in volume in the process of fermentation, and the center of the pellets was autolysis due to the lack of nutrient delivery, which extremely reduced the yield of polysaccharides. Therefore, it is necessary to excavate a fermentation strategy based on morphological regulation for *Paraisaria dubia* to promote polysaccharides accumulation.

**Results:**

In this study, we developed a method for enhancing polysaccharides production by *Paraisaria dubia* using microparticle enhanced technology, talc microparticle as morphological inducer, and investigated the enhancement mechanisms by transcriptomics. The optimal size and dose of talc were found to be 2000 mesh and 15 g/L, which resulted in a high polysaccharides yield. It was found that the efficient synthesis of polysaccharides requires an appropriate mycelial morphology through morphological analysis of mycelial pellets. And, the polysaccharides synthesis was found to mainly rely on the ABC transporter-dependent pathway revealed by transcriptomics. This method was also showed excellent robustness in 5-L bioreactor, the maximum yields of intracellular polysaccharide and exopolysaccharides were 83.23 ± 1.4 and 518.50 ± 4.1 mg/L, respectively. And, the fermented polysaccharides were stable and showed excellent biological activity.

**Conclusions:**

This study provides a feasible strategy for the efficient preparation of cordyceps polysaccharides via submerged fermentation with talc microparticles, which may also be applicable to similar macrofungi.

**Graphical Abstract:**

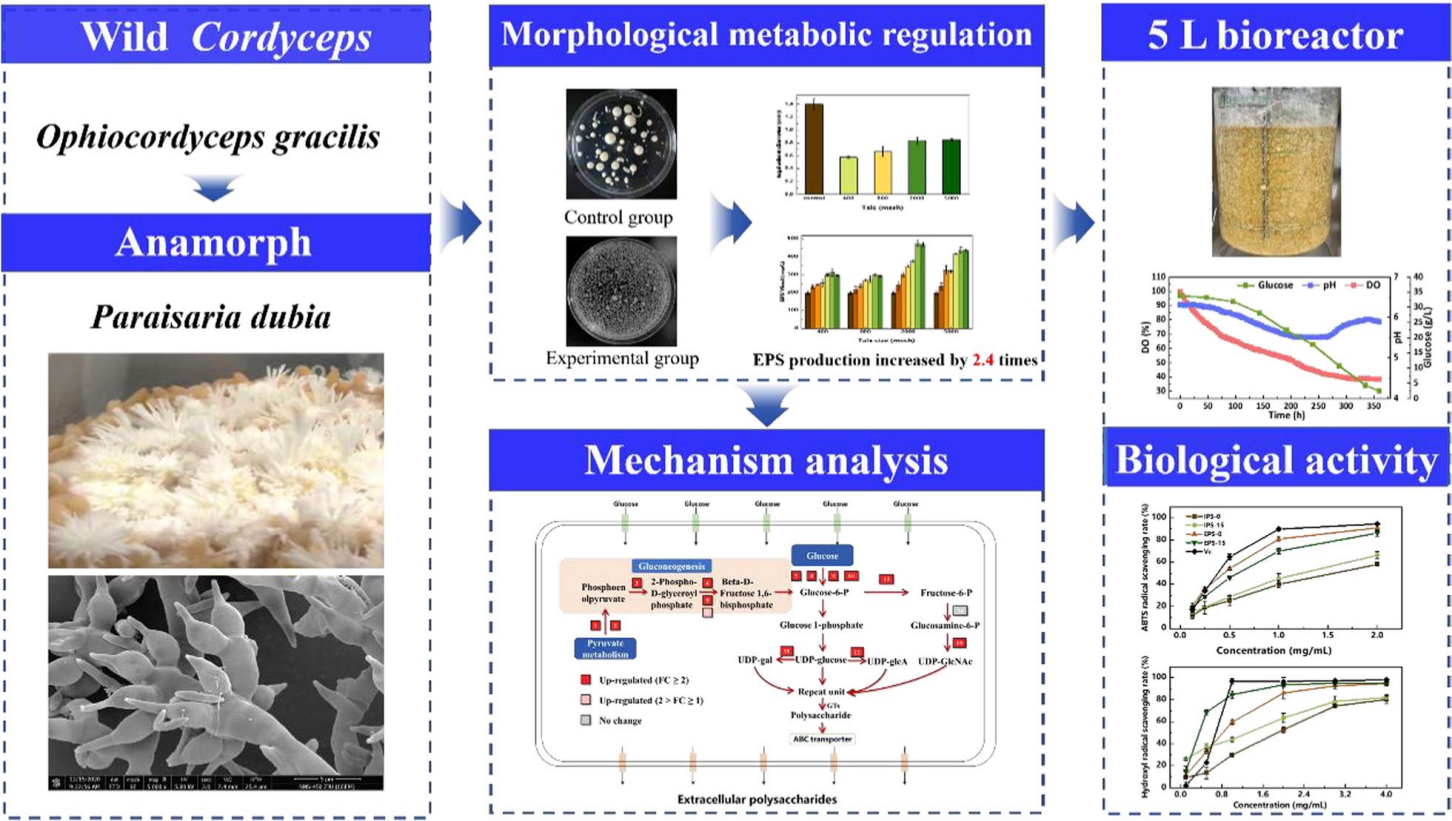

**Supplementary Information:**

The online version contains supplementary material available at 10.1186/s12934-021-01733-w.

## Introduction

*Cordyceps* has been used as a kind of Chinese herbology and tonic for hundreds of years, and about 750 species have been identified to date [1]. Cordyceps polysaccharides (CPs) are important active ingredients in *Cordyceps* and have many physiological functions, such as anti-tumor, immunomodulatory, antioxidation, anti-inflammatory, anti-aging, and anti-fatigue effects [2, 3]. However, it is particularly difficult to obtain such polysaccharides because *Cordyceps* has become an endangered species due to overharvesting from the wild. The technology of bionic ecological cultivation may be a good choice to solve this dilemma [4]. Nevertheless, *Cordyceps* has a long growth cycle, the production of fruiting bodies strongly depends on environmental conditions, and cultured fruiting bodies have a low biological conversion rate, resulting in a particularly low yield of CPs in culture [5]. Therefore, the search for superior alternative technologies to obtain sufficient CPs has become an advanced research hotspot.

The formation of natural C*ordyceps* fruiting bodies is a fascinating biological process. The mycelia of *Cordyceps* infects the parasitifer (larvae or pupae of lepidopteran insects) and causes it to be ossified, after which the macrofungus grows out of the sporangium under suitable conditions, thus forming a complex structure with a stalk (the visible part, also known as the fruit body) and the sclerotium (the infected corpse of the insect) [6, 7]. Studies have shown that polysaccharides and other active ingredients in natural *Cordyceps* are mainly give the credit to these macrofungi [2]. The isolation of these macrofungi and deep liquid fermentation may be an effective way to obtain CPs [4]. Similar methods for obtaining secondary metabolites have been applied to macrofungi such as *Ganoderma lucidum*, *Lentinus edodes* and *Hericium erinaceus*, mainly for the production of polysaccharides, terpenoids, nucleosides and other active substances [8–10]. In our previous research, *Ophiocordyceps gracilis*, an important species of *Cordyceps*, was obtained and its anamorph (*Paraisaria dubia*, *P. dubia*) was also isolated and cultivated. The active ingredients obtained from the fermentation of *P. dubia* were similar to natural C*ordyceps*, especially polysaccharides [11]. However, the mycelial pellets of *P. dubia* were dense and increased in volume as the fermentation progressed, and the pellet center was prematurely autolyzed duo to difficulties of nutrient delivery, which greatly reduced the synthesis efficiency of polysaccharides. Therefore, it is necessary to develop a fermentation strategy based on morphological regulation for *P. dubia* to promote polysaccharide accumulation.

In macrofungi, morphology control is a common problem affecting the synthesis of target products in liquid fermentation [12]. Various strategies have been applied to achieve efficient synthesis of target products through morphological control of macrofungi [13]. These strategies are mainly based on physical and chemical methods, such as controlling the inoculum size, mechanical stress (aeration, stirring), pH, glass beads, surfactants, and other factors [14, 15]. The maximum exopolymer yield of 2.33 g/L was achieved in *Grifola frondosa* when its morphology was controlled by changing the aeration rate and agitation [16]. The concentration of 2-mercaptohistidine trimethyl betaine increased approximately three times due to the change of morphological type in shiitake (*Lentinula edodes*) [17]. L-phenylalanine can also change the morphology of *Ganoderma lucidum*, leading to decreased cell wall thickness and increased porosity, which increased the production of exopolysaccharides (EPS) to 0.91 g/L after a 24 h culture [18]. Macrofungal morphology affects the rheology of the fermentation broth, mass transfer and aeration during the fermentation process [19]. Furthermore, the membrane (wall) structure was also reconstructed with the change of macrofungal morphology, thus enhancing nutrient absorption and the release of target products [20]. Additionally, the cellular molecular metabolic network will also be rearranged and promote the synthesis of products [21, 22]. However, this regulation strategy is specific for different macrofungi, and there was little research on the synthesis of polysaccharides through fermentation of *P. dubia*. Accordingly, there was no reasonable morphological control method to achieve efficient synthesis of polysaccharides by *P. dubia*. The relationship between mycelial pellet morphology and polysaccharides synthesis, as well as the molecular mechanisms controlling the production of CPs was not clear.

In this study, we developed a method for improving polysaccharides production by *P. dubia* using talc, and the enhancement mechanisms were investigated at different levels. Firstly, the fermentation characteristics and polysaccharides synthesis were explored in the presence of talc microparticles with different sizes and doses, and the relationship between morphology and high yield of polysaccharides was revealed on the macro level. Secondly, the expression of key genes and enzymes in the polysaccharide synthesis pathway of *P. dubia* was analyzed on the transcriptional level, and the metabolic network was deduced using bioinformatic methods. Thirdly, the robustness of the high yield strategy for polysaccharide production was investigated in a 5-L bioreactor. Finally, the biological activity of the polysaccharides produced under the optimized conditions was assessed using an in vitro antioxidant assay. This study provides rational guidance for the large-scale fermentation of *P. dubia* to efficiently produce CPs, which may also be applicable to similar macrofungi.

## Results and discussion

### Effects of talc microparticles on the fermentation characteristics of *P. dubia* affect

**Fig. 1:**
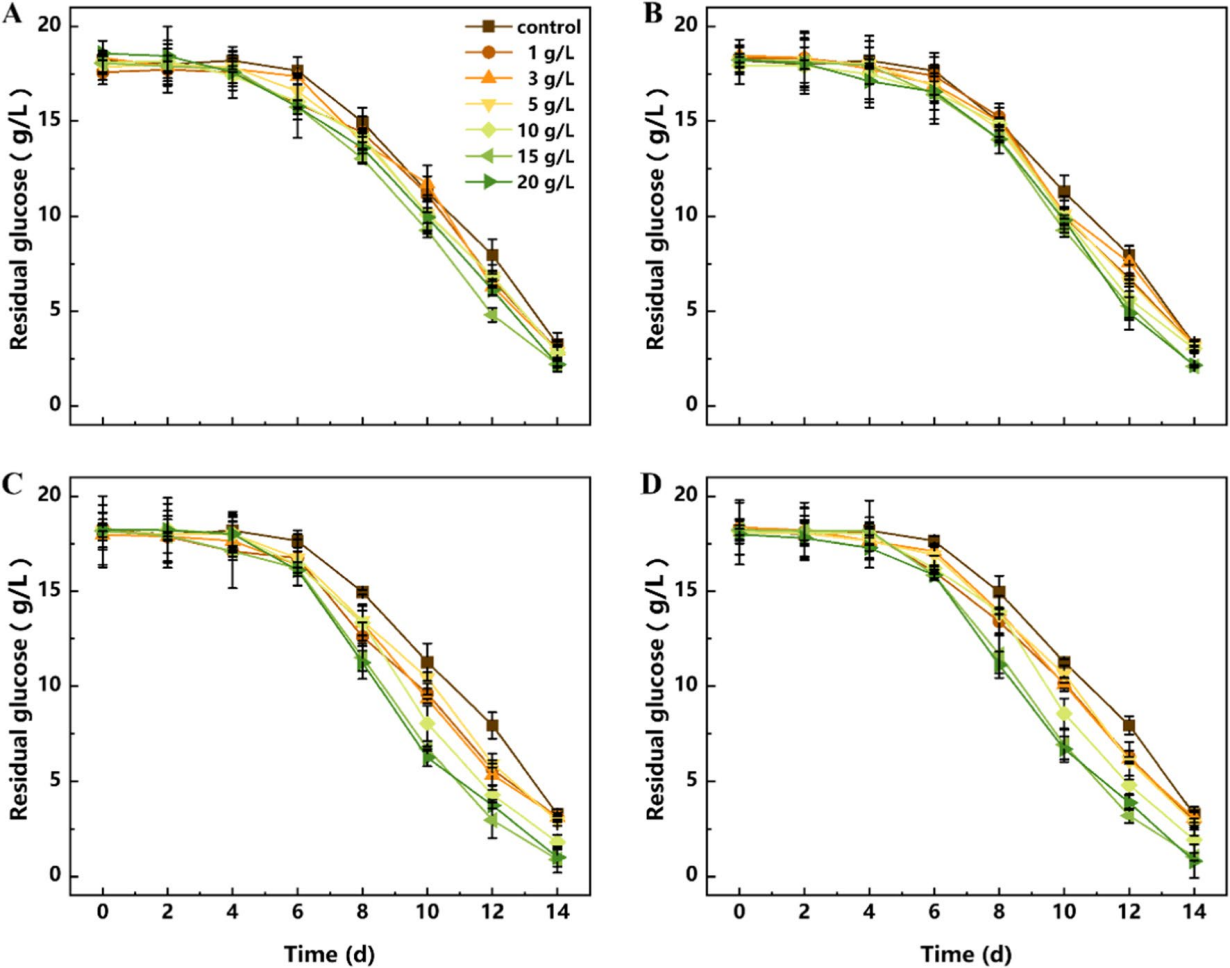
Effects of talc with different particle sizes on the substrate consumption of *P. dubia*. **a** 400 mesh; **b** 800 mesh; **c** 2000 mesh; **d** 5000 mesh

**Fig. 2:**
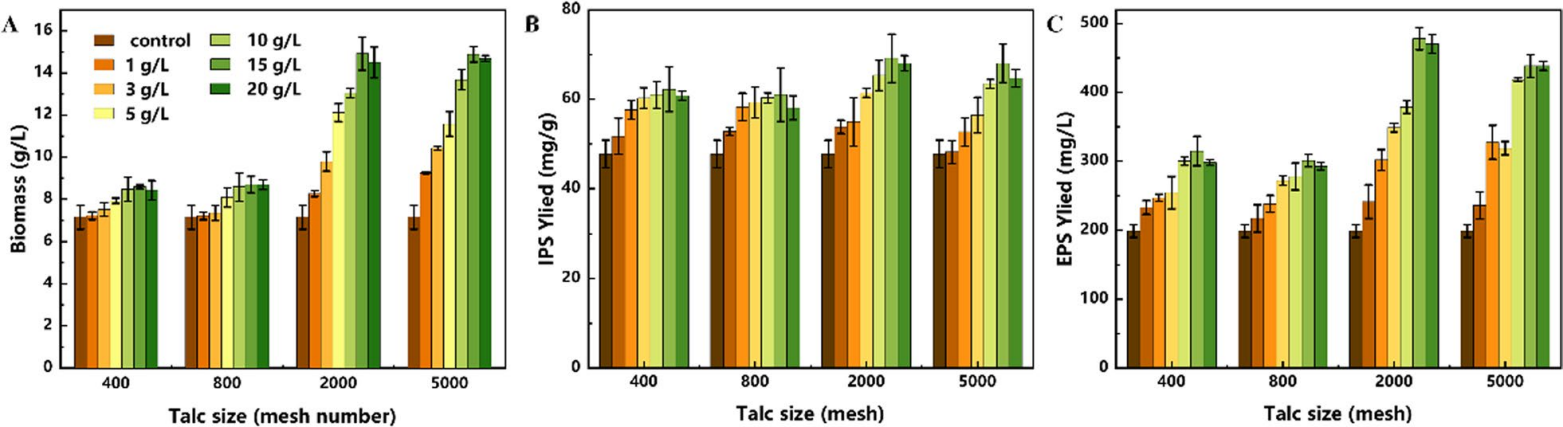
The effects of talc on the biomass, intracellular polysaccharides (IPS) and exopolysaccharides (EPS) of *P. dubia*. **a** Biomass; **b** IPS; **c** EPS

In order to explore the effects of talc microparticles on the physiological characteristics of *P. dubia*, the substrate consumption, biomass and polysaccharide synthesis were investigated in liquid fermentations with different doses of talc particles with different sizes. As shown in Figs. 1 and 2, substrate consumption, biomass accumulation and polysaccharide synthesis were the lowest in the control group. The reason is that large mycelial pellets in the control group may contain inactive or dead cells in the inner structure due to limited nutrient transfer. With the addition of talc, the fermentation characteristics showed an increasing trend, and the growth effect was most obvious when the size of talc particles was small (2000 mesh and 5000 mesh). When the size of talc particles was large (400 mesh and 800 mesh), the growth effect was much less obvious. This may be due to the strong grinding effect of large talc particles in the fermentation mixture, which is not conducive to the growth and reproduction of mycelial pellets. In addition, this enhancement of substrate consumption and biomass accumulation did not persist with the increase of talc dose, and the effect was most significant in the treatment groups supplemented with 2000 and 5000 mesh talc at different doses. As shown in Figs. 1C, D and 2A, in the treatment groups with 2000 and 5000 mesh talc, the substrate consumption and biomass accumulation continued to increase until the concentration reached 15 g/L, and the maximum biomass (14.92 ± 0.78 g/L) was obtained when 2000 mesh talc was added with 15 g/L. However, these two indexes showed a decreasing trend when the talc concentration reached 20 g/L. This phenomenon is similar to a study on the production of natural yellow pigments in *Monascus purpureus* using morphological regulation of mycelial pellets based on microparticles [23]. This result clearly indicated that macrofungi have complex responses to the type and dosage of exogenous additives (even inert substances) during liquid fermentation. Accordingly, *P. dubia* also had an optimal response to the size and dose of talc, and the addition of 15 g/L talc at 2000 mesh resulted in the best growths.

Polysaccharides are the core active ingredient of *P. dubia*, and the effect of talc on polysaccharide synthesis is shown in Fig. 2B and C. The effect of talc on polysaccharides synthesis was similar to the effect on biomass accumulation. Both intracellular polysaccharides (IPS) and EPS responded positively to talc addition and their yields were both increased compared with the control group. Among them, the highest yield of IPS reached 69.16 ± 5.4 mg/g when 15 g/L of 2000 mesh talc was added. By contrast, 62.22 ± 5.1, 61.05 ± 6.3, and 68.03 ± 4.3 mg/g of IPS was produced under the optimal concentration of talc with a size of 400 mesh, 800 mesh, and 5000 mesh, respectively. The synthesis efficiency of EPS was higher when 2000 and 5000 mesh talc was added than with 400 and 800 mesh talc. The highest yield of EPS was also obtained at 15 g/L of 2000 mesh talc, reaching 478.20 ± 16.3 mg/L. This was 150.6% and 158.9% higher than with 400 mesh and 800 mesh talc, respectively. One of the possible reasons may be that the biomass of mycelia grown with 15 g/L of 2000 mesh talc was higher than under the other conditions. Another possibility was that the addition of talc makes the mycelium pellets reach the optimal form of polysaccharides synthesis, and the changes in mycelium pellets morphology were also observed during fermentation. The previous studies have also reported the correlation between mycelium pellet morphology and active ingredients synthesis [24]. In spite of this, the substrate consumption rate, cell growth and polysaccharide synthesis were significantly improved after talc was added, but it is unclear how mycelial pellet morphology and fungal metabolism was regulated by talc.

### The correlation between mycelial morphology and polysaccharide synthesis of *P. dubia* affected by talc

In macrofungi, mycelial pellet morphology was reported to be closely correlated with the production of polysaccharides [24]. In this study, the mycelial morphology of *P. dubia* was also significantly changed when talc was added, which may be related to the massive synthesis of polysaccharides. In order to study the correlation between mycelial pellet morphology and polysaccharides synthesis, the morphological parameters of mycelial pellets and polysaccharides synthesis in fermentations containing 15 g/L of talc with different sizes were recorded and compared.

**Fig. 3:**
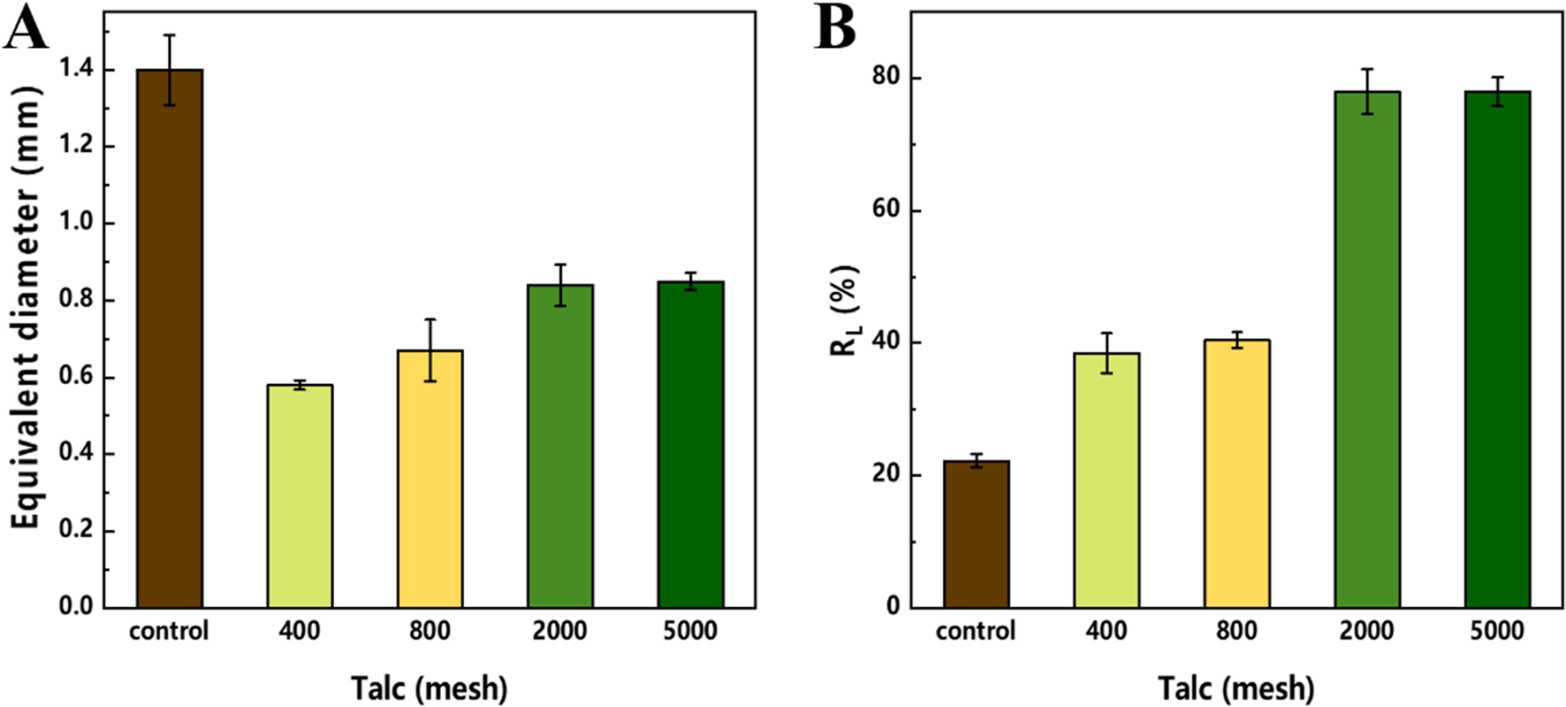
Morphological parameters of *P. dubia* mycelial pellets grown with talc of different sizes. **a** equivalent diameter; **b** ratio of the loose layer of the pellet to the total size

The equivalent diameter of mycelial pellets with talc of different sizes is summarized in Fig. 3A. Following the addition of talc, the equivalent diameter of mycelial pellets was significantly reduced, which indicates that talc has the effect of changing the mycelial pellet morphology of *P. dubia*, especially at 400 and 800 meshes. However, the optimal biomass and polysaccharides yield were obtained with 2000 mesh talc (Figs. 1 and 2). It is possible that the larger size of talc is not conducive to the growth and metabolism *P. dubia*. Talc has an irregular shape and sharp edges, so that it can collide with mycelial pellets and shear mycelia during fermentation [14]. In particular, the collision and shear forces are more intense when the size of talc is larger. This phenomenon was also reflected in Fig. 3B. The ratio of the loose layer in mycelial pellets increased as the size of talc decreased, indicating that the mycelial pellets had large and dense nuclei in the control group, which limited material transfer and restricted polysaccharide synthesis. The large size of talc changed the morphology of the mycelial pellets, but its sharp edges inhibited the growth of the loose layer. The talc with 2000 and 5000 mesh not only changed the morphology of mycelial pellets but also promoted the increase of loose layers, which can be attributed to the lower shearing with the smaller particle size. Moreover, the small size of talc particles can provide a carrier for the growth and winding of mycelia, so that the mycelial pellets become a complex of mycelium and talc, which were looser and enhanced the nutrient transfer, thus realizing the efficient accumulation of biomass and polysaccharides. However, this effect did not increase as the talc size decreased. The equivalent diameter and loose layer ratio of mycelial pellets were very similar under the conditions of talc with 2000 and 5000 mesh. These results showed that the mycelial pellet morphology can be effectively controlled and polysaccharide synthesis can be significantly improved when talc with a size of 2000 mesh was used.

**Fig. 4:**
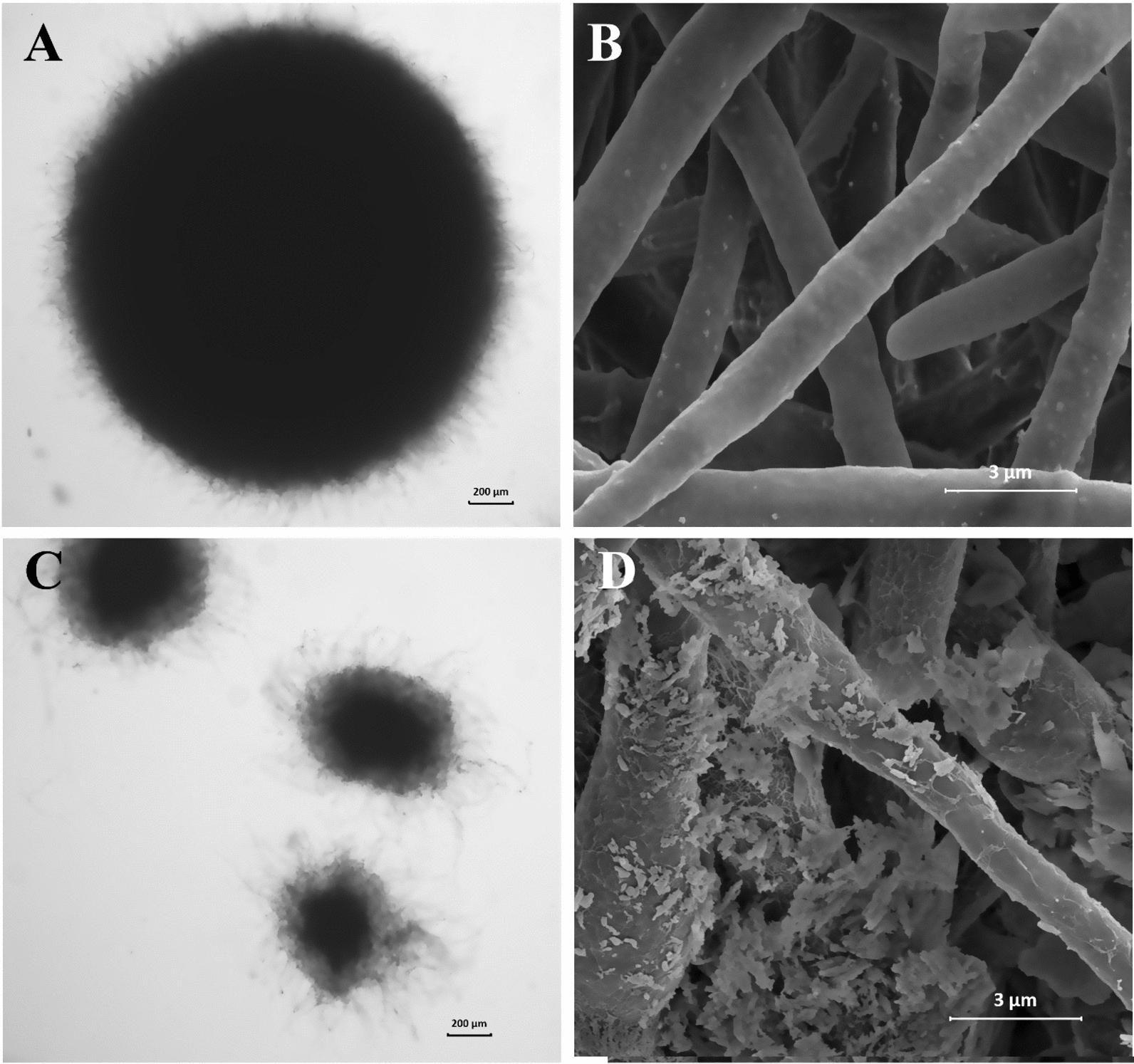
Typical morphological changes of *P. dubia* following the addition of talc. **a** and **c** control; **b** and **d** group with talc

As shown in Fig. 4, the mycelial pellet morphology changes resulting from supplementation with 15 g/L of 2000 mesh talc were observed by optical microscopy and scanning electron microscopy (SEM). It is clear that exogenous addition of these compounds brought about great changes in mycelial pellet morphology. In detail, the core layer of mycelial pellets was small and exhibited a loose structure with the addition of 15 g/L of 2000 mesh talc (Fig. 4A and C), which was in contrast to the control group and was consistent with the morphological parameters of mycelial pellets mentioned above. The SEM images in Fig. 4B and D show that the control mycelia were smooth and rounded, while the mycelia grown with the addition of 15 g/L of 2000 mesh talc were rough and irregular. Under these conditions, the mycelial surface structure was changed and the secretion of substances from the mycelial surface was increased, so that polysaccharides and glycoproteins were produced in response to the sharp edges of talc, which is consistent with previous reports [25]. Therefore, talc can significantly change the morphology and structure of mycelia and promote the exchange and transfer of nutrients and products, resulting in the efficient synthesis of polysaccharides. In particular, when 15 g/L of 2000 mesh talc was added, the mycelial pellets developed an optimal morphology for biomass accumulation and synthesis of CPs. This may be an effective strategy to achieve efficient synthesis of polysaccharides if it can be replicated on the bioreactor scale.

### Investigation of the polysaccharide synthesis mechanism of *P. dubia*

**Fig. 5:**
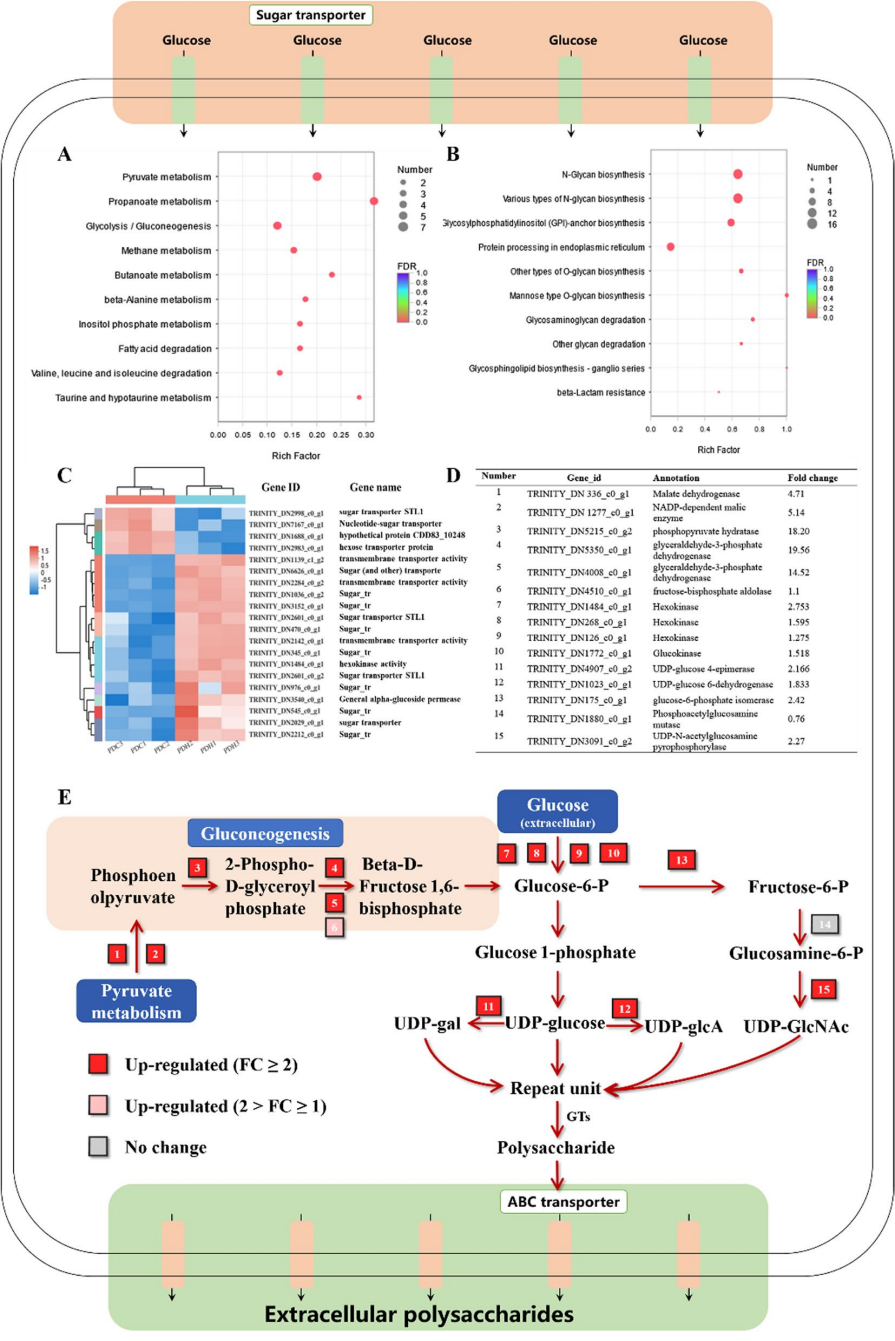
Pathways related to polysaccharide synthesis in *P. dubia*.  **a** KEGG enrichment analysis of genes related to carbohydrate metabolism; **b** KEGG enrichment analysis of genes related to glycan biosynthesis; **c** Heatmap analysis of genes related to sugar transporters; **d** Genes and enzymes involved in the polysaccharide metabolism of *P. dubia* (DEGs related to the pathways shown in Fig. 5E); **e** Putative pathway for polysaccharide biosynthesis

In order to investigate the enhancement mechanisms of polysaccharides synthesis, transcriptomics was used to analysis the expression of key genes and enzymes in polysaccharides synthesis pathway of *P. dubia*, enabling us to reconstruct the metabolic network. The verification of RNA-seq results was carried out by qPCR to confirm the reliability of the RNA-seq data. Samples were collected from the experimental group (15 g/L of 2000 mesh talc) and the control group. The differentially expressed genes (DEGs) were identified using the criteria |log2 (fold change) |> 1 and q-value < 0.05 as the threshold, as shown in Additional file 1: Fig S1. COG and GO analysis showed that these DEGs are mainly concentrated in the category carbohydrate transport and metabolism as well as metabolic process (Additional file 1: Figs. S2 and S3). Carbohydrates are the key to the synthesis of complex glycoconjugates. Therefore, we preformed KEGG enrichment analysis of DEGs related to carbohydrate metabolism and polysaccharide synthesis, which showed that the related DEGs are mainly concentrated in pyruvate metabolism, propanoate metabolism, gluconeogenesis/glycolysis, and various types of N-glycan biosynthesis (Fig. 5A and B). It is conceivable that accelerated cell growth and polysaccharide biosynthesis under this strategy requires a considerable energy supply and active metabolism. The coordination of these pathways may be the key to increasing the production of polysaccharides. The biosynthesis of polysaccharides is a complex process and the related reactions can be divided into three parts according to the currently available information, including the synthesis of sugar nucleotides, assembly of the repeat units, and finally polymerization and export [26]. Further analysis of DEGs related to these three pathway steps was carried out, which identified 322 gens, accounting for approximately 9% of the total DEGs. These included genes related to monosaccharide transport, pyruvate metabolism, gluconeogenesis, assembly of glycosyl donors and ABC transporters.

In the bacterial polysaccharide synthesis pathway, sugar nucleotides are the most basic glycosyl donor unit, and their efficient supply is the key to achieving the mass synthesis of polysaccharides [27, 28]. Monosaccharides, mostly glucose in this study, need to undergo a series of reactions after they enter the cell to be converted into the glycosyl donor unit [29]. In these reactions, glucose-6-phosphate is a key metabolite that acts as a link between different up- and downstream pathways. It can be converted into glucose-1-phosphate, fructose-6-phosphate by phosphoglucomutase, glucose-6-phosphate isomerase, and then glucose-1-phosphate to generate UDP-glucose (the glycosyl donor unit of polysaccharides structure) by uridylyltransferase [30]. UDP-glucose can also be further converted into other glycosyl donors, such as UDP-Gal and UDP-GlcA [31, 32]. In addition, fructose-6-phosphate can be converted into UDP-N-acetylglucosamine (UDP-GlcNAc) by UDP-N-acetylglucosamine pyrophosphorylase [33]. In agreement with the hypothesis, the enzymes associated with these transformation steps were among the upregulated DEGs in the mycelia grown with talc compared to the control gorup. In particular, UDP-glucose 6-dehydrogenase, UDP-glucose 4-epimerase and UDP-N-acetylglucosamine pyrophosphorylase showed an increase in their respective transcription levels by 1.83-, 2.16- and 2.27-fold, respectively. These three enzymes can convert UDP-glucose and glucosamine-6-phosphate into UDP-Gal, UDP-GlcA and UDP-GlcNAc (Fig. 5D and E). In addition, glucose-6-phosphate can also enter the glycolysis pathway to produce pyruvate, which can further be used aerobically pathway in the citric acid cycle to produce ATP and other products, providing energy for polysaccharide synthesis [34]. Notably, the enzymes involved in the anabolism of glucose-6-phosphate showed varying degrees of upregulation in the experimental group. Heatmap analysis of the identified DEGs showed that genes associated with transmembrane transport of monosaccharides clustered together, and most were also upregulated (Fig. 5C). These include sugar transporters (TRINITY_DN6626_c0_g1, TRINITY_DN1036_c0_g1, TRINITY_DN3152_c0_g1, TRINITY_DN470_c0_g1, TRINITY_DN345_c0_g1, TRINITY_DN976_c0_g1, TRINITY_DN545_c0_g1, TRINITY_DN2212_c0_g1), and a putative transporter (TRINITY_DN3152_c0_g1), which are primarily anchored to the cell membrane. The transmembrane transport of monosaccharides marks the first step of microbial metabolism and the beginning of glucose-6-phosphate synthesis. After glucose enters the cell, it can be converted into glucose-6-phosphate by hexokinase and glucokinase (TRINITY_DN1484_c0_g1, TRINITY_DN1772_c0_g1), which were also respectively upregulated 2.75- and 1.6-fold.

Moreover, the KEGG enrichment pathway analysis of carbohydrate metabolism showed that genes related to gluconeogenesis and pyruvate metabolism were significantly upregulated in the experimental group (Fig. 5A and Additional file 1: Table S1). These two pathways are linked together, leading to the production of glucose-6-phosphate, which means that their upregulated could promote the production of glucose-6-phosphate and further provide the intermediates for polysaccharide synthesis (Fig. 5E). These results indicated that talc addition not only changed the appearance of mycelia, but also changed the cell membrane protein composition and metabolic capacity, which promoted the transmembrane transport of glucose and enhanced the synthesis of glycosyl donor units.

Many different glycosyl donors are synthesized in cytoplasm, but they cannot be polymerized without initial glycosyltransferases (GTs), which are conserved among different fungi [35]. This initial step is similar to the synthesis of bacterial lipopolysaccharide O-antigen, a typical model system of polysaccharide biosynthesis [30]. At the beginning of O-antigen biosynthesis, sugar nucleotides are used as the sugar donors, and the lipid carrier undecenoyl phosphate (Und-P) located on the cell membrane is the sugar receptor, which is activated by an initiating GT to form Und-PP-linked glycoconjugates [28]. Subsequently, the polysaccharide chain is assembled via different pathway mechanisms, including the Wzy polymerase-dependent pathway, ABC transporter-dependent pathway, and synthase-dependent pathway [14, 30]. In our study, transcripts related to the ABC transporter-dependent pathway were subjected to KEGG pathway enrichment analysis in the experimental group. A total of 120 genes related to the ABC transporter-dependent pathway were identified, 58.3% of which were upregulated (Additional file 1: Tables S2–4). Therefore, it can be inferred that polysaccharide synthesis in *P. dubia* mainly depends on the ABC transporter-dependent pathway. In this pathway, glycosyl donor units were assembled by the action of GTs at the cytoplasmic face of the inner membrane. When only a single GT-containing operon is involved the synthesis results in homopolymers, and when multiple GTs are used for the assembly process, they can produce heteropolymers. These polymers are then further modified by glycosyl hydrolases (GHs) until the mature chain is produced [28, 36]. In our study 14 genes encoding GTs were upregulated in the experimental group compared to the control group. These GTs were annotated to 8 families, and 31 upregulated GH genes belonging to 20 families were identified by transcriptome data. GT90 can modify the sugar chain of *Cryptococcus neoformans*, which plays a very important role in the production of novel polysaccharides [37]. In addition, most of the cell-secreted β-glucans are synthesized by GT2 family enzymes present in ascomycetes and basidiomycetes [38, 39]. During the high-yield production of *Ganoderma* polysaccharides, there is significant upregulation of GH gene expression, which is considered to be directly related to the yield of polysaccharides [29, 40]. As can be seen from the above results, it can be inferred that the polysaccharides synthesized by *P. dubia* are structurally variable heteropolymers. The mature polysaccharides are exported across the inner membrane and translocated to the cell surface by ABC transporters. In our study, there were 8 significantly upregulated DEGs related to ABC transporters in the experimental group compared to the control group (Additional file 1: Table S2). The upregulated DEGs related to ABC transporters (TRINITY_DN4624_c0_g2, TRINITY_DN7253_c0_g1, TRINITY_DN6508_c0_g1, TRINITY_DN226_c0_g2, TRINITY_DN3551_c0_g2, TRINITY_DN2963_c0_g1, TRINITY_DN1668_c0_g1, TRINITY_DN491_c0_g1) indicated that talc could regulate the phenotypic and physiological responses of *P. dubia*, thus further adapting the cells to environmental changes and providing sufficient nutrient transport for mycelial growth and EPS biosynthesis (Fig. 5D and E). These may be one of the key factors affecting polysaccharide synthesis.

Overall, integrated analysis of gene expression and gene end-products associated with polysaccharide synthesis between the experimental group and the control group revealed that the relationship between mycelial morphology and glycosyl donor formation forms an important basis for polysaccharide synthesis, and the ABC transporter-dependent pathway (GTs, GHs, and ABC transporters) may be the key to efficient polysaccharide synthesis under this fermentation strategy.

### Scale-up of the fermentation strategy in a 5-L bioreactor

The key criterion to evaluate the feasibility of a new fermentation strategy is an initial scale-up to a laboratory bioreactor. To test this, *P. dubia* was grown in a 5-L bioreactor with 15 g/L of 2000 mesh talc.

**Fig. 6:**
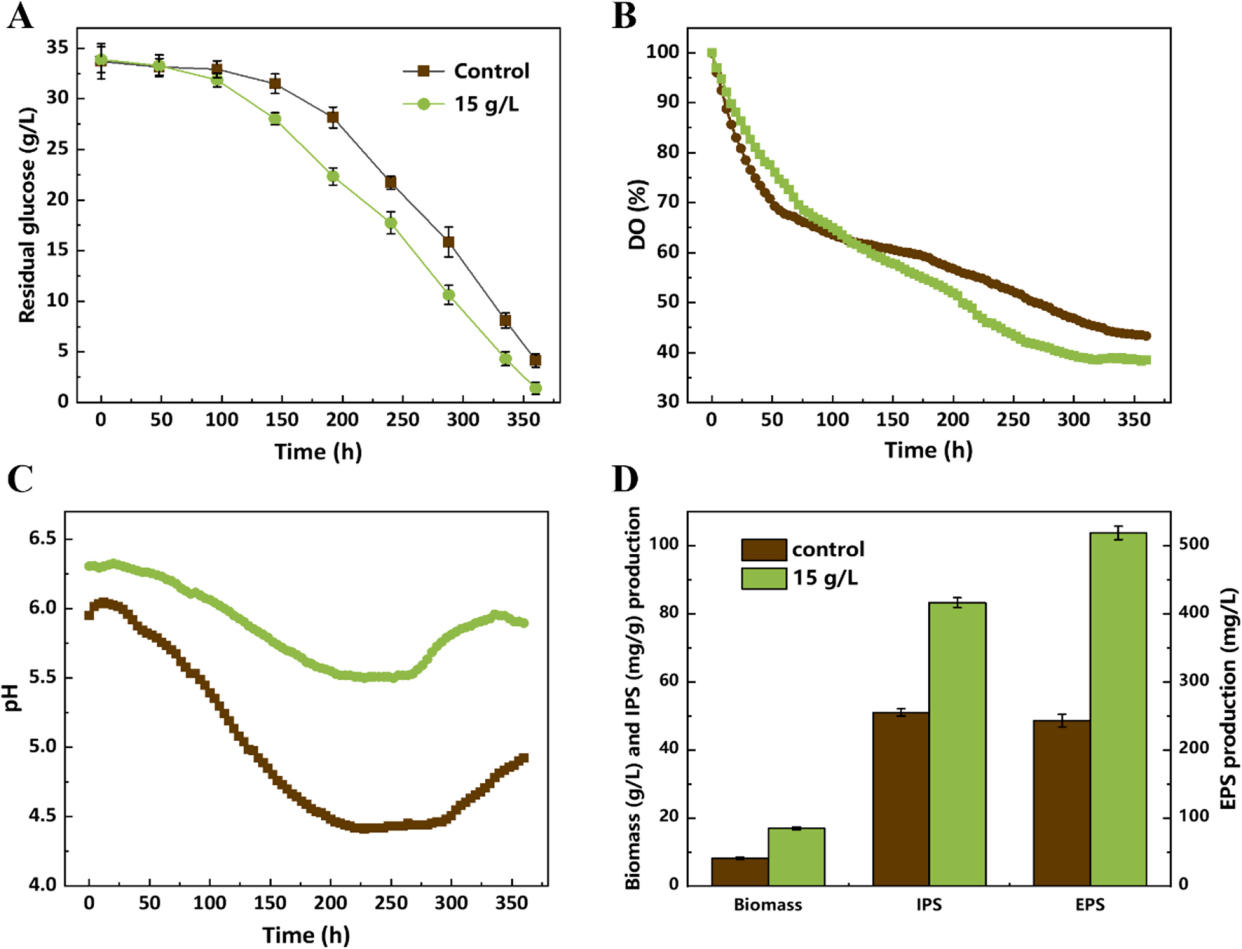
The fermentation characteristics of *P. dubia* in a 5-L bioreactor. **a** Residual glucose (g/L); **b** DO (%); **c** pH; **d** Biomass, IPS and EPS production

As shown in Fig. 6, the *P. dubia* maintained stable growth characteristics under this strategy, and the fermentation characteristics were similar to the results of shake-flask experiments. After a lag period, the rate of glucose consumption increased significantly, and was significantly higher in the experimental group than in the control group without talc (Fig. 6A). The density of mycelial pellets was changed after adding talc particles, and the equivalent diameter of mycelial pellets was also reduced, which may have improved the mass transfer capacity of mycelial pellets. This phenomenon was also reflected in the dissolved oxygen concentration (DO) curve (Fig. 6B). The DO showed a continuous decrease throughout the fermentation process due to the normal growth of mycelial pellets, which consume oxygen. The demand for oxygen increased significantly in the experimental group after 96 h of culture. The pH curve shown in Fig. 6C depicts the trend of continuous decrease in the early fermentation period and subsequent increase. This phenomenon mirrors the sequential metabolism of the nitrogen sources and the production of organic acids, which can induce a reduction of the pH. However, the overall pH level of the experimental group was significantly higher than that of the control group, which may indicate that talc has a certain pH buffering effect. As shown in Fig. 6D, the biomass, IPS and EPS yields of the experimental grou reached 16.97 ± 0.21 g/L, 83.23 ± 1.4 mg/mL and 518.50 ± 4.1 mg/L, respectively, representing 2.07-, 1.6- and 2.16-fold improvements compared with the control, respectively. Taken together, these results confirm that the superior fermentation performance of this strategy can be scaled up, and it has the potential for industrial application.

### Bioactivity assessment of polysaccharides produced by *P. dubia*

The biological activity is the most valuable characteristic of CPs. In other studies of macrofungi, the biological activity of polysaccharides exhibited large deviations when the fermentation strategy was changed [20]. In order to verify the bioactivity of polysaccharides obtained by fermenting *P. dubia* in a 5-L bioreactor, the antioxidant activity, an important indicator of biological activity, was measured following the isolation of intracellular and EPS (IPS-15, EPS-15) from the talc supplemented group and control group (IPS-0, EPS-0).

**Fig. 7:**
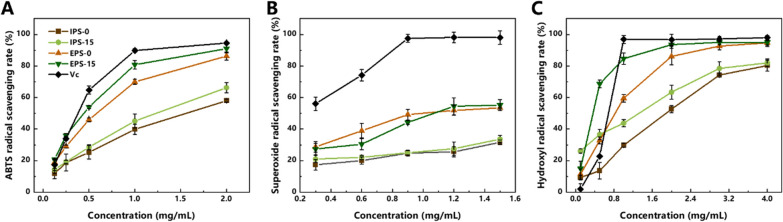
The free radical scavenging capacity of polysaccharides obtained from *P. dubia* grown using different cultivation strategies. **a** ABTS radical scavenging rate (%); **b** superoxide radical scavenging rate scavenging rate (%); **c** hydroxyl radical scavenging rate (%); IPS-0 and EPS-0: Intracellular polysaccharides and exopolysaccharide from the control culture without talc; IPS-15 and EPS-15: Intracellular polysaccharides and exopolysaccharide from the experimental group with 15 g/L of 2000 mesh talc

As shown in Fig. 7, the scavenging activities of ABTS, superoxide anions and hydroxyl radicals were determined, and compared to vitamin C as a positive control. In all three free radical scavenging assays, IPS and EPS showed superior biological activity, which exhibited a dose–response relationship. Furthermore, EPS generally exhibited a higher free radical scavenging ability than IPS. This is consistent with reports in the related literature, which may be due to structural differences of EPS and IPS. Furthermore, the scavenging ability of IPS-15 was slightly higher than that of the control (IPS-0) in all three radical scavenging assays. For EPS, the ABTS radical and superoxide anion radical scavenging abilities of EPS-0 and EPS-15 were similar, and increased with the polysaccharide concentration (Fig. 7A and B). Notably, the ability of EPS-15 to scavenge hydroxyl radicals was significantly higher than that of EPS-0, especially at a polysaccharide concentration of 0.5 mg/mL. The hydroxyl radical ability of EPS-15 was 2.1-fold higher than that of EPS-0 (Fig. 7C). Thus, the use of talc did not interfere with the product quality. According to the above results, the fermentation strategy proposed in this study has strong industrialization potential, and can maintain stable antioxidant activity of CPs while increasing the product yield.

## Conclusions

When 15 g/L of 2000 mesh talc was added as a morphological inducer to promote the growth of mycelial pellets, the yield of IPS and EPS in shake flasks reached 69.16 ± 5.4 mg/g and 478.20 ± 16.3 mg/L, respectively. Talc effectively changed the morphology of the mycelium and promoted the transfer of nutrients and products. The efficient polysaccharide synthesis was ascribed to a synergistic relationship between glycosyl donor synthesis and the ABC transporter-dependent pathway. The highest yields of IPS and EPS in a 5 L bioreactor reached 83.23 ± 1.4 mg/mL and 518.50 ± 4.1 mg/L, respectively, and the resulting polysaccharides showed high antioxidant activity.

## Materials and methods

### Strain and culture conditions

*P. dubia* HL-119 (CGMCC No.20731) was obtained from China General Microbiological Culture Collection Center (CGMCC), and was preserved in 20% (v/v) glycerol at − 80 °C. The culture medium contained (per liter): glucose 20 g/L, peptone 10 g/L, KH_2_PO_4_ 2 g/L, and MgSO_4_·7H_2_O 0.5 g/L. The seed culture (10% v/v) was grown in fermentation shake flasks with 50 mL medium at 20 °C and 120 rpm until the glucose was completely consumed. For the fermentation, 300 mL of seed culture was used to inoculate a 5-L bioreactor (Shanghai T&J Bio-engineering, China) containing 3 L of fermentation medium. The impeller speed was set to 150 rpm and the temperature was set to 20 °C. Samples were collected in regular intervals, and a bioanalyzer (SBA-40C, Institute of Biology, Shandong Academy of Sciences, China) was used to measure the glucose levels enzymatically.

### Analytical methods

#### Determination of biomass

The fermentation broth (100 mL) was collected every 2 days, and the mycelial pellets were washed 3 times with ultrapure water, then freeze-dried and weighed. Biomass was defined as the total mycelium weight minus the mass of added talc.

#### Extraction and content determination of polysaccharides

The extraction of intracellular and EPS of *P. dubia* was conducted as described before [41], with minor modifications as follows. Mycelium powder after grinding was extracted two times with ultrapure water (1:20, w/v) at 80 °C for 3 h. The combined filtrate and fermentation broth were concentrated in a rotary evaporator under reduced pressure. The mycelium filtrate and fermentation broth were precipitated with a four-fold volume of 95% ethanol at 4 °C for 16 h, and centrifuged three times at 7871 × g for 15 min to obtain IPS and EPS. Then, the total polysaccharide content was determined using the phenol–sulfuric acid method [34].

### Morphological analysis of mycelial pellets

The morphological analysis was based on previous literature [42], with minor modifications as follows. The equivalent diameter of mycelial pellets was analyzed using Image-Pro Plus 6.0 software (Media Cybernetics Inc., MD, USA), and derived from the area (A) as equivalent diameter (D) = $\sqrt {4 \times A/\pi }$. The loose layer and a dense layer were divided according to the form of mycelial pellets in the liquid fermentation, and the proportion of the loose layer in the whole pellet was calculated according to the formula:

$${\text{R}}_{{\text{L}}} = \, \left( {{\text{R}}_{{{\text{max}}}} - {\text{ R}}_{{{\text{core}}}} } \right) \, /{\text{ R}}_{{{\text{max}}}}$$

where R_L_ is the ratio of the loose layer in mycelial pellets, R_max_ is the maximal mean radius of the mycelial pellets, and R_core_, is the radius of the dense core of mycelial pellets.

SEM observation of mycelial pellet morphology was conducted in analogy to a reported method for the mycelial pellets of *Monascus purpureus* [23]. The surface of the sample was sputtered with a thin layer of gold, and the surface shape was observed by a field emission scanning electron microscope (SU8010 Tokyo, Japan).

### RNA sequencing

After the samples with 15 g/L talc (PDH1, PDH2, PDH3) and control (PDC1, PDC2, PDC3) were harvested on the ninth day, the TruSeqTM RNA Sample Preparation Kit (Illumina, San Diego, CA) was used to prepare samples for RNA sequencing. RNA purification, reverse transcription, library construction and sequencing were performed at Shanghai Majorbio Bio-pharm Biotechnology Co., Ltd. (Shanghai, China) according to the manufacturer’s instructions (Illumina, San Diego, CA). Qubit2.0 and agarose gel electrophoresis were used to determine the RNA concentration, RNA integrity and genomic DNA contamination. The TPM (Transcripts Per Million) method was used to calculate gene expression levels. We sued the DESeq2 R package to perform differential expression analysis for pairs of conditions/groups. Benjamini and Hochberg's method was used to adjust the obtained q values to control for the false discovery rate. DEGs were identified based on the threshold of q value < 0.05 and | log2 (fold change) |> 1. The clusterProfiler R package was used to assess the statistical enrichment of DEGs in KEGG and GO pathways.

### Antioxidant properties

#### Determination of ABTS radical-scavenging ability

The ABTS radical scavenging assay was based on a published method [43], with minor modifications as follows. A 7 mmol/L ABTS working stock solution was prepared, mixed with an equal volume of 2.45 mmol/L potassium persulfate solution and placed it in the dark environment for one week at 4 °C. The ABTS working solution was diluted 50 times with anhydrous ethanol, after which 1.0 mL polysaccharide sample solution (0, 0.125, 0.25, 0.5, 1.0, 2.0 mg/mL) was mixed with 2.0 mL of the diluted ABTS working solution, and the reaction was allowed to proceed in the dark for 6 min. Then, the absorbance at 734 nm was measured and recorded as A_1_. The solution containing no polysaccharides was used as the negative control, and the absorbance was recorded as A_0_. With vitamin C as a positive control, the ABTS radical scavenging rate was calculated using the formula ABTS radical scavenging rate (%) = (1 − A_1_/A_0_) × 100.

#### Determination of superoxide anion radical scavenging ability

The superoxide anion radical scavenging activity of *P. dubia* polysaccharides was determined using a published method [44]. Briefly, 1 mL of the polysaccharide solution (0.3, 0.6, 0.9, 1.2, or 1.5 mg/mL) was mixed with 4.5 mL 50 mmol/L Tris–HCl (pH 8.2) and 2.4 mL ultrapure water, and the mixture was reacted in a water bath at 25 °C for 10 min. Then, 0.3 mL of a 45 mmol/L pyrogallol solution were added, allowed to react for 4 min, and stopped by adding 0.5 mL hydrochloric acid. After reacting for 10 min, the absorbance of the mixture at 325 nm was recorded as A_1_. vitamin C and the solution without polysaccharides were the positive and the negative controls, respectively, and the absorbance was recorded as A_0_. The calculation formula for scavenging superoxide anion free radicals by polysaccharides was as follows: Scavenging rate (%) = (1-A_1_/A_0_) × 100.

#### Determination of hydroxyl radical scavenging ability

The hydroxyl radical activity was measured based on previous reports [43, 45]. Briefly, 1 mL of the polysaccharide solution (0.1, 0.5, 1.0, 2.0, 3.0, or 4.0 mg/mL) was mixed with 1.0 mL of an FeSO_4_ solution (2 mmol/L) and 1.0 mL salicylic acid–ethanol solution (6 mmol/L). Then, 1.0 mL H_2_O_2_ (6 mmol /L) was added to the mixture and incubated in a water bath at 37 °C for 30 min. The absorbance at 510 nm was recorded as A_1_ with vitamin C as the positive control. The absorption value of the sample group and control with ultrapure water instead of H_2_O_2_ water were recorded as A_0_ and A_2_, respectively. The calculation formula of hydroxyl radical scavenging by polysaccharides was as follows: hydroxyl radical scavenging rate (%) = [1 − (A_1_ − A_2_)]/(A_0_) × 100.

### Quantitative real-time PCR analysis

RT-PCR validation was conducted by Shanghai Majorbio Bio-pharm Biotechnology Co., Ltd. (Shanghai, China), and 4 genes were randomly selected for RT-PCR to verify the RNA-seq data.

### Statistical analysis

All experiments were performed in triplicates. The data were expressed as means ± standard deviations (n = 3). Data sets were evaluated by one-way analysis of variance.

## Supplementary Information

**Additional file 1: Table S1**. Significantly different genes related to gluconeogenesis and pyruvate metabolism. **Table S2**. Significantly upregulated genes related to ABC transporter. **Table S3**. Significantly different genes related to GTs. **Table S4**. Significantly different genes related to glycosylhydrolases (GHs).** Fig S1**. Statistics of the difference in the expression of DEGs in P. dubia. **Fig S2**. COG functional classification histogram of DEGs in P. dubia. **Fig S3**. GO classification of DEGs in P. dubia.

## Data Availability

All data generated or analyzed during this study are included in this published article and its additional files.

## Abbreviations

CPs: Cordyceps polysaccharides
IPS: Intracellular polysaccharides
EPS: Exopolysaccharides
SEM: Scanning electron microscopy
DEGs: Differentially expressed genes
UDP-GlcNAc: UDP-N-acetylglucosamine
GTs: Glycosyltransferases
GHs: Glycosyl hydrolases
DO: Dissolved oxygen
