# Supplementary material for: Enhancement of polysaccharides production using microparticle enhanced technology by Paraisaria dubia
Source: Microb Cell Fact. 2022 Jan 28;21:12. doi: 10.1186/s12934-021-01733-w (PMC8796560; doi:10.1186/s12934-021-01733-w)
Supplement: Supplementary file 1 — Additional file 1: Table S1. Significantly different genes related to gluconeogenesis and pyruvate metabolism. Table S2. Significantly upregulated genes related to ABC transporter. Table S3. Significantly different genes related to GTs. Table S4. Significantly different genes related to glycosylhydrolases (GHs). Fig S1. Statistics of the difference in the expression of DEGs in P. dubia. Fig S2. COG functional classification histogram of DEGs in P. dubia. Fig S3. GO classification of DEGs in P. dubia. [file 12934_2021_1733_MOESM1_ESM.doc]

**Development a** **method for improving** **cordyceps polysaccharide production by *Paraisaria dubia* using microparticle enhanced technology**

Ling-Ling Tong ^1#^, Yue Wang ^1#^, Li Yuan ^1^, Meng-Zhen Liu ^1^, Yuan-Hang Du ^1^, Xin-Ya Mou ^1^, Qing-Hao Yang ^1^, Shi-Xiang Wei, Jun-Ya Li ^1^, Mian Wang ^1^, Dong-Sheng Guo ^1*^

1 School of Food Science and Pharmaceutical Engineering, Nanjing Normal University, NO 1, Wenyuan Road, Nanjing 210023, People’s Republic of China.

*Corresponding authors: Tel: +86 25 58139942; Fax: +86 25 58139389

E-mail: [guodongs@njnu.edu.cn](mailto:guodongs@njnu.edu.cn)

**Supplementary tables and figures**

**Table S1.** Significantly different genes related to gluconeogenesis and pyruvate metabolism.

**Table S2.** Significantly upregulated genes related to ABC transporter.

**Table S3.** Significantly different genes related to glycosyltransferases (GTs).

**Table S4.** Significantly different genes related to glycosylhydrolases (GHs).

**Fig. S1.** Statistics of the difference in the expression of DEGs in *P. dubia*.

**Fig. S2.** COG functional classification histogram of DEGs in *P. dubia*.

**Fig. S3.** GO classification of DEGs in *P. dubia*.

**Table S1.** Significantly different genes related to gluconeogenesis and pyruvate metabolism.

| Gene_ID | NR description | Fold change | Pvalue |
| --- | --- | --- | --- |
| TRINITY_DN1716_c0_g1 | hypothetical protein XA68_16395 | 1.00 | 0.961988471258 |
| TRINITY_DN1108_c0_g1 | phosphoenolpyruvate synthase | 4.10 | 1.31501008744E-7 |
| TRINITY_DN175_c0_g1 | glucose-6-phosphate isomerase | 2.42 | 7.49687732843E-25 |
| TRINITY_DN3348_c0_g1 | pyruvate decarboxylase | 10.96 | 4.09618493017E-7 |
| TRINITY_DN1277_c0_g1 | NADP-dependent malic enzyme | 5.142 | 5.97336E-62 |
| TRINITY_DN1108_c0_g1 | phosphoenolpyruvate synthase | 4.103 | 1.31501E-07 |
| TRINITY_DN6372_c0_g1 | acetyl-CoA C-acetyltransferase | 2.065 | 4.38168E-34 |
| TRINITY_DN104_c0_g1 | acetate kinase | 2.042 | 3.98754E-27 |
| TRINITY_DN2913_c0_g1 | hypothetical protein CDD83_4731 | 1.939 | 1.60497E-26 |
| TRINITY_DN417_c1_g1 | acetyltransferase component of the pyruvate dehydrogenase complex, partial | 1.803 | 3.76208E-19 |
| TRINITY_DN2897_c0_g1 | fumarate hydratase, mitochondrial, partial | 1.697 | 2.4845E-13 |
| TRINITY_DN2244_c0_g1 | malate synthase, partial | 1.538 | 5.5479E-12 |
| TRINITY_DN1038_c0_g1 | hypothetical protein DL763_010678 | 0.482 | 6.62375E-24 |
| TRINITY_DN2506_c0_g1 | hypothetical protein HIM_08098 | 0.475 | 3.6582E-39 |
| TRINITY_DN1402_c0_g1 | hypothetical protein TOPH_01782 | 0.412 | 6.07173E-55 |

**Table S2.** Significantly upregulated genes related to ABC transporter.

| Gene_ID | NR description | Fold change | | Pvalue |
| --- | --- | --- | --- | --- |
| TRINITY_DN4624_c0_g2 | ATP-binding cassette sub-family A member 3 | 19.492 | 0.009085293 | |
| TRINITY_DN7253_c0_g1 | unnamed protein product, partial | 15.278 | 0.239995931 | |
| TRINITY_DN6508_c0_g1 | hypothetical protein CDD80_3927 | 6.541 | 0.012773279 | |
| TRINITY_DN226_c0_g2 | ABC transporter G family member 11 | 2.774 | 3.60464E-26 | |
| TRINITY_DN3551_c0_g2 | ABC transporter CDR4 | 2.077 | 4.24473E-16 | |
| TRINITY_DN2963_c0_g1 | ABC transporter CDR4 | 2.045 | 8.71844E-05 | |
| TRINITY_DN1668_c0_g1 | canalicular multispecific organic anion transporter 2 | 1.94 | 2.88021E-13 | |
| TRINITY_DN491_c0_g1 | multidrug resistance protein 1B, partial | 1.914 | 1.25053E-21 | |
| TRINITY_DN1457_c0_g1 | [NU+] prion formation protein 1 | 1.562 | 5.90654E-13 | |
| TRINITY_DN5739_c0_g2 | ABC transporter ced-7 | 1.561 | 0.003040569 | |
| TRINITY_DN1543_c0_g1 | ATP-binding cassette transporter abc4 | 1.506 | 5.46979E-17 | |
| TRINITY_DN3991_c0_g1 | oligomycin resistance ATP-dependent permease | 1.454 | 1.35882E-13 | |
| TRINITY_DN3533_c0_g1 | ribosome biogenesis protein bms1 | 1.45 | 1.51818E-08 | |
| TRINITY_DN120_c0_g1 | hypothetical protein CDD83_8155 | 1.41 | 3.01856E-17 | |
| TRINITY_DN2906_c0_g1 | multidrug resistance protein 1A | 1.402 | 0.002877003 | |

**Table S2. continue**

| Gene_ID | NR description | Fold change | Pvalue |
| --- | --- | --- | --- |
| TRINITY_DN6259_c0_g1 | ATP-dependent bile acid permease | 1.302 | 0.000669437 |
| TRINITY_DN1242_c0_g1 | heavy metal tolerance protein | 1.206 | 0.000216465 |
| TRINITY_DN3157_c0_g1 | ATP-dependent bile acid permease | 1.199 | 0.004546165 |
| TRINITY_DN3836_c0_g1 | putative ABC transporter ATP-binding protein/permease | 1.19 | 0.036626274 |
| TRINITY_DN2669_c0_g1 | canalicular multispecific organic anion transporter 1 | 1.164 | 0.108014321 |
| TRINITY_DN2483_c0_g1 | putative ABC transporter ATP-binding protein/permease | 1.081 | 0.763205033 |
| TRINITY_DN1526_c0_g1 | metal resistance protein YCF1 | 1.078 | 0.112178527 |
| TRINITY_DN2416_c0_g1 | peroxisomal long-chain fatty acid import protein 2 | 1.035 | 0.442895552 |
| TRINITY_DN2911_c0_g1 | ABC transporter CDR4 | 1.02 | 0.835052813 |
| TRINITY_DN4682_c0_g2 | ABC transporter | 1.015 | 0.960756455 |
| TRINITY_DN7362_c0_g1 | hypothetical protein DL763_009662 | 1 | 1 |

**Table S3.** Significantly different genes related to glycosyltransferases (GTs).

| Family | Gene_ID | Fold change | Pvalue |
| --- | --- | --- | --- |
| GT1 | TRINITY_DN1851_c0_g1 | 1.975 | 3.80404E-15 |
| GT1 | TRINITY_DN1773_c1_g1 | 1.975 | 7.35181E-18 |
| GT1 | TRINITY_DN350_c0_g1 | 1.269 | 8.81183E-06 |
| GT | TRINITY_DN3967_c0_g1 | 1.442 | 1.4455E-09 |
| GT2 | TRINITY_DN3443_c0_g1 | 4.163 | 3.18466E-62 |
| GT2 | TRINITY_DN3323_c0_g1 | 1.274 | 1.55854E-06 |
| GT2 | TRINITY_DN1002_c0_g1 | 1.16 | 0.002683832 |
| GT2 | TRINITY_DN2670_c0_g1 | 1.306 | 5.06322E-09 |
| GT2 | TRINITY_DN6352_c0_g1 | 0.268 | 1.49697E-26 |
| GT15 | TRINITY_DN2573_c0_g1 | 1.295 | 1.01533E-05 |
| GT20 | TRINITY_DN3284_c0_g1 | 1.647 | 2.93449E-14 |
| GT20 | TRINITY_DN481_c0_g4 | 1.534 | 6.38568E-11 |
| GT28 | TRINITY_DN1253_c0_g1 | 1.158 | 0.001195409 |
| GT34 | TRINITY_DN1217_c0_g1 | 1.733 | 1.73493E-20 |
| GT90 | TRINITY_DN5188_c0_g2 | 8.444 | 0.012184114 |
| GT90 | TRINITY_DN2888_c0_g2 | 0.405 | 2.33356E-36 |

**Table S4.** Significantly different genes related to glycosylhydrolases (GHs).

| Family | Gene_ID | Fold change | Pvalue |
| --- | --- | --- | --- |
| GH | TRINITY_DN2432_c0_g1 | 1.154 | 0.000500456 |
| GH127 | TRINITY_DN1831_c0_g1 | 2.128 | 7.00538E-26 |
| GH15 | TRINITY_DN2687_c0_g1 | 1.513 | 1.25365E-10 |
| GH16 | TRINITY_DN1952_c0_g1 | 5.913 | 2.5463E-18 |
| GH16 | TRINITY_DN2340_c0_g1 | 4.589 | 4.58529E-40 |
| GH16 | TRINITY_DN6846_c0_g1 | 3.997 | 2.06782E-15 |
| GH16 | TRINITY_DN4428_c0_g1 | 3.484 | 1.74631E-06 |
| GH16 | TRINITY_DN1093_c0_g1 | 1.55 | 7.05338E-06 |
| GH16 | TRINITY_DN1319_c0_g1 | 1.449 | 5.28276E-11 |
| GH16 | TRINITY_DN6255_c0_g1 | 0.823 | 0.000689702 |
| GH17 | TRINITY_DN78_c0_g1 | 1.517 | 1.50734E-09 |
| GH17 | TRINITY_DN1434_c0_g1 | 1.139 | 0.016144844 |
| GH2 | TRINITY_DN3278_c0_g1 | 2.347 | 2.19396E-22 |
| GH2 | TRINITY_DN2517_c0_g1 | 2.325 | 8.00481E-26 |
| GH2 | TRINITY_DN7194_c0_g1 | 1.437 | 4.10924E-06 |
| GH2 | TRINITY_DN2303_c0_g1 | 1.169 | 0.003953613 |
| GH20 | TRINITY_DN7171_c0_g1 | 1.581 | 5.49426E-07 |

**Table S4. continue**

| Family | Gene_ID | Fold change | Pvalue |
| --- | --- | --- | --- |
| GH28 | TRINITY_DN3369_c0_g2 | 2.973 | 3.6732E-08 |
| GH3 | TRINITY_DN428_c0_g1 | 2.262 | 1.22009E-08 |
| GH3 | TRINITY_DN2727_c0_g1 | 1.859 | 7.76343E-05 |
| GH3 | TRINITY_DN428_c1_g1 | 1.854 | 2.17946E-05 |
| GH3 | TRINITY_DN859_c0_g1 | 0.827 | 0.000512553 |
| GH32 | TRINITY_DN1223_c0_g2 | 1.334 | 0.001822421 |
| GH47 | TRINITY_DN1631_c0_g1 | 1.744 | 5.55215E-08 |
| GH47 | TRINITY_DN3141_c0_g1 | 1.396 | 0.000163283 |
| GH47 | TRINITY_DN280_c0_g3 | 1.319 | 0.010303572 |
| GH47 | TRINITY_DN593_c1_g1 | 1.182 | 0.000440751 |
| GH5 | TRINITY_DN651_c0_g1 | 1.5 | 2.70E-10 |
| GH63 | TRINITY_DN1210_c0_g1 | 1.17 | 0.001430946 |
| GH72 | TRINITY_DN1847_c0_g1 | 2.734 | 8.64274E-15 |
| GH76 | TRINITY_DN85_c0_g1 | 1.303 | 9.82408E-05 |
| GH76 | TRINITY_DN4183_c0_g1 | 1.283 | 0.00105586 |
| GH76 | TRINITY_DN2290_c0_g1 | 0.24 | 7.49578E-11 |
| GH92 | TRINITY_DN5082_c0_g1 | 1.714 | 3.81085E-21 |

**Fig S1.** Statistics of the difference in the expression of DEGs in *P. dubia*.

**Fig S2.** COG functional classification histogram of DEGs in *P. dubia*. (A) RNA processing and modification; (B) Chromatin structure and dynamics; (C) Energy production and conversion; (D) Cell cycle control, cell division, chromosome partitioning; (E) Amino acid transport and metabolism; (F) Nucleotide transport and metabolism; (G) Carbohydrate transport and metabolism; (H) Coenzyme transport and metabolism; (I) Lipid transport and metabolism; (J) Translation, ribosomal structure and biogenesis; (K) Transcription; (L) Replication, recombination and repair; (M) Cell wall/membrane/envelope biogenesis; (O) Posttranslational modification, protein turnover, chaperones; (P) Inorganic ion transport and metabolism; (Q) Biosynthesis, transport and catabolism of secondary metabolites; (T) Signal transduction mechanisms; (U) Intracellular trafficking, secretion, and vesicular transport; (V) Defense mechanisms; (Z) Cytoskeleton.


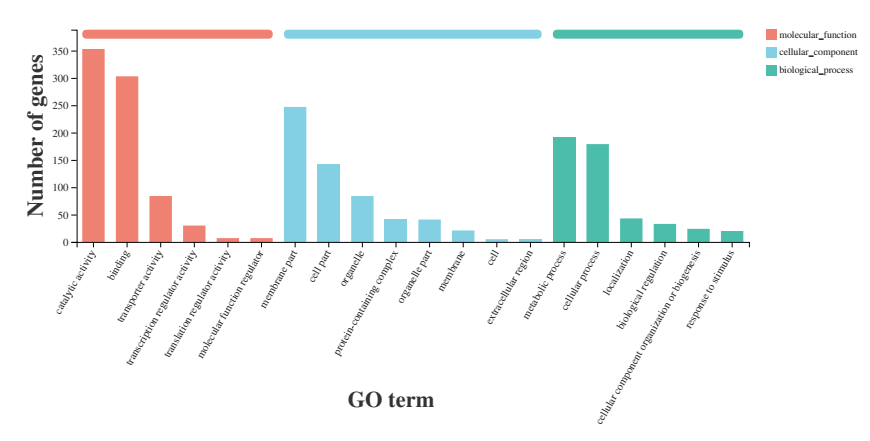


**Fig S3.** GO classification of DEGs in *P. dubia*.
